# Association of changes in overall and specific leisure activities with long-term all-cause mortality in older adults: a nationwide cohort study

**DOI:** 10.7189/jogh.15.04119

**Published:** 2025-05-16

**Authors:** Chi Zhang, Anying Bai, Yuting Kang, Yushan Zhang, Jianliang Zhao, Hong Shi, Ji Shen

**Affiliations:** 1 The Key Laboratory of Geriatrics, Beijing Institute of Geriatrics, Institute of Geriatric Medicine, Chinese Academy of Medical Sciences, Beijing Hospital, National Center of Gerontology of National Health Commission, Beijing, China; 2 School of Population Medicine and Public Health, Chinese Academy of Medical Sciences and Peking Union Medical College, Beijing, China; 3 Department of Science Research, Beijing Hospital, National Center of Gerontology, Institute of Geriatric Medicine, Chinese Academy of Medical Sciences, Beijing, China; 4 Department of Geriatrics, Beijing Hospital, National Center of Gerontology, Institute of Geriatric Medicine, Chinese Academy of Medical Sciences, Beijing, China; 5 School of Medical Humanities, Peking University Health Science Center, Beijing, China

## Abstract

**Background:**

Leisure activities (LAs) are vital for healthy ageing and are linked to lower mortality risk in older adults. However, most previous longitudinal studies have assessed LAs at only one time point. We aimed to explore the impact of dynamic changes in LAs on subsequent all-cause mortality among older adults.

**Methods:**

We enrolled 21 262 older adults who had participated in the six waves of the Chinese Longitudinal Healthy Longevity Survey (CLHLS) in 1998, 2000, 2002, 2005, 2008, and 2011. All participants completed two consecutive assessments of LAs (including seven typical activities) during the first two waves (mean interval = 2.72 years (standard deviation = 0.08)); we further followed them up until 2018, *i.e.* beyond the 2011 CLHLS. We divided them into five categories according to the change pattern of LAs: stable low (low-low), stable moderate (moderate-moderate), stable high (high-high), LAs increase (low-moderate, low-high, moderate-high), and LAs decrease (high-moderate, high-low, moderate-low). We used a Cox proportional hazard model to test the association between changes in LAs and all-cause mortality, including demographic characteristics, health behaviours, and disease history as covariates.

**Results:**

We documented 15 065 death events during 80 045.39 person-years of follow-up. Compared with the stable moderate group, the adjusted hazard ratios (aHRs) of mortality for the stable low group and stable high group were 1.27 (95% confidence interval (CI) = 1.21–1.35) and 0.66 (95% CI = 0.62–0.71), respectively. An increase in LAs was associated with a lower risk of mortality (aHR = 0.83; 95% CI = 0.78–0.88), while a decrease in LAs was associated with a higher risk of mortality (aHR = 1.05; 95% CI = 1.01–1.09). The protective effect of LAs increase on premature death was more pronounced in men than in women. The main results remained stable in subgroup and sensitivity analyses.

**Conclusions:**

Maintaining and increasing participation in leisure time activities significantly reduced the risk of all-cause mortality in community-dwelling older individuals in our sample.

According to data from China's seventh national population census in 2020, around 13.5% of the country’s total population or some 190 million individuals were aged ≥65 years [1]. Projections indicate that by 2030, this proportion will exceed 20%, signalling a substantial demographic shift toward an aged society [2]. Engagement in recreational activities such as physical exercise, intellectual endeavours, and social interactions has been shown to positively affect mental health and cognitive functions, aligning with a proactive approach to healthy longevity. However, whether sustained or increased engagement in leisure activities (LAs) can further reduce mortality risk remains inadequately explored in the context of the older Chinese population. As ageing progresses, the health status and living conditions of the older population continue to change, underscoring the need to examine changes in LAs patterns and their health implications.

A wealth of domestic and international longitudinal research has demonstrated that active participation in LAs reduces the risk of disability, cognitive decline, and chronic diseases in older adults, with a notable inverse correlation to mortality rates [3–5]. For example, Byberg and colleagues [6] and Schnohr and colleagues [7] found that maintaining or increasing the degree of LAs was associated with a lower risk of death, though their focus was primarily on activities prevalent in Western countries. Similarly, two cohort studies from China [4,8] reported that frequent engagement in LAs may reduce the risk of disability and mortality in the oldest-old Chinese population. In previous studies conducted in Western or European countries, engagement in common LAs such ball games (like tennis and golf) and other outdoor sports (like hiking and cycling) was reported to benefit older adults [9,10]. These activities are often designed to promote physical fitness and social interaction in environments with well-developed infrastructure and recreational facilities. However, such activities may not be readily accessible or suitable for the social and environmental contexts of communities in China. Moreover, older individuals in China, a populous low- and middle-income developing country, may favour more group-oriented and productive activities that can be easily adapted to local conditions and resources. Therefore, when examining the impact of specific types of LAs on healthy ageing, it is essential to fully consider their adaptability to regional cultural diversity. Besides, most previous observational studies in China have typically measured LAs at a single point in time, overlooking the evolving nature of these behaviours. They have also often used relatively small sample sizes, limiting their generalisability.

In general, LAs encompass various physical, cognitive, and productive activities, but evidence on their associations with all-cause mortality remains mixed [4,11]. Physical LA has been shown to reduce the risk of cardiovascular disease [12], cancer [13] and mortality [14], while sedentary behaviours are often linked to higher risks of chronic diseases and mortality [15,16]. However, some sedentary LAs, such as cognitive and cultural activities, have been associated with positive health outcomes [17,18]. Huang and colleagues [19] found that maintaining or increasing physical LA levels lowers mortality risk, While Glass and colleagues [20] demonstrated that participation in social and productive activities reduces mortality among older adults. Similarly, frequent social engagement has been linked to lower mortality risk [21]. However, most studies have focussed on either physical LA or social activities, without systematically comparing the effects of various LAs on all-cause mortality. Furthermore, evidence on how different LAs reduce mortality risk among older Chinese adults remains scarce.

Here we retrieved data from the Chinese Longitudinal Healthy Longevity Survey (CLHLS) to analyse changes in LAs among community-dwelling older adults and their long-term association with all-cause mortality risk. Additionally, we aimed to examine how changes in different types of specific LAs may have distinct mechanisms influencing mortality risk.

## METHODS

### Study population

We included newly enrolled participants from the first five waves (1998, 2000, 2002, 2005, 2008, and 2011) of the CLHLS. Participants were required to have completed at least three follow-up surveys. The frequency of LA participation was assessed twice consecutively during the first two waves using standardised scales, while survival information was collected in subsequent follow-ups. The initial cohort consisted of 43 583 individuals, excluding 130 individuals aged under 60 and 36 individuals with missing data on LAs in the first wave. During the second wave, 13 086 participants had died and 5946 were lost to follow-up. Among the remaining 24 385 survivors, 46 individuals were missing LA data, resulting in a final sample of 24 339 individuals for follow-up. During the study period, 2994 additional participants were lost to follow-up and 83 had invalid death dates, resulting in a final sample of 21 262 participants (Figure S1 in the **Online Supplementary Document**). This study was based on publicly available data from the CLHLS and is thus exempt from requiring ethical approval. Approval for the CLHLS project was obtained from the Biomedical Ethics Committee of Peking University (IRB00001052-13074), and informed consent was obtained from all participants or their guardians.

### Measurement and change of LAs

**Figure 1:**
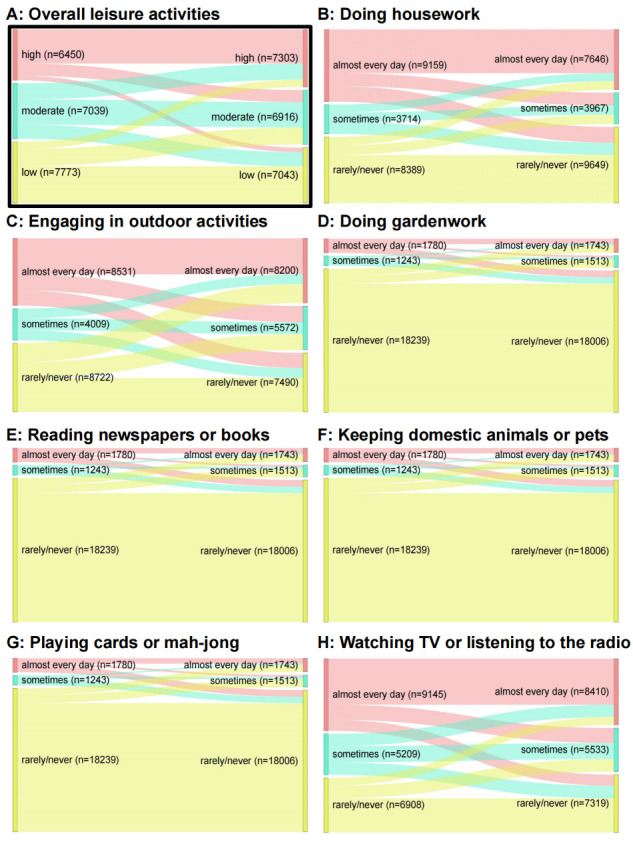
Changing patterns of overall and specific LAs in the total sample. **Panel A.** Changing patterns of the overall LAs. **Panels B–H.** Changing patterns of specific types of LAs.

Participants’ engagement in seven typical LAs (reading newspapers or books, outdoor activities, housework, gardening, keeping domestic animals or pets, playing cards or mahjong, and watching television or listening to the radio) was assessed through face-to-face interviews. The frequency of participation in each activity was categorised as ‘almost every day’ (2 points), ‘sometimes’ (1 point), or ‘never’ (0 points) (Table S1 in the **Online Supplementary Document**). The total LAs score ranged from 0 to 14, with higher scores reflecting more frequent engagement in leisure activities. The LAs scale was designed to be culturally appropriate and user-friendly for Chinese older adults. Its Cronbach’s alpha coefficient in our study was 0.866, indicating high internal consistency reliability. Based on tertiles, we categorised LAs into three levels: high (9–14), moderate (5–8), and low (0–4). We determined changes in LAs by comparing the differences between two consecutive measurements (mean interval = 2.72 years (standard deviation = 0.08)). We further classified participants into five groups based on changes in their LAs scores across two assessments: stable low (low-low, n = 4329), stable moderate (moderate-moderate, n = 3200), stable high (high-high, n = 4666), LAs increase (low-moderate, low-high, and moderate-high, n = 3919), and LAs decrease (high-moderate, high-low, and moderate-low, n = 5148) (**Figure 1**).

### Survival outcomes and time

The CLHLS team obtained survival data through local civil affairs systems or by contacting next of kin during follow-ups, with the time of death recorded. For deceased individuals, we defined survival time as the interval between inclusion and death; for participants who were alive or censored, we calculated it as the interval between the last valid follow-up and the time of inclusion. Participants lost to follow-up after the first assessment were excluded from the analysis, as we could not determine accurate survival times.

### Covariates

Based on prior studies [3,4,7,19,22], we sequentially adjusted our multivariable analysis for the following covariates: age, sex, household registration (rural or urban), ethnicity (Han or other), financial support type (self-supported through employment, retirement wage, or relative/community), family income (adequate or not), living alone, education level (literacy or illiteracy), body mass index, marital status (spouse alive or not), smoking, and alcohol consumption. Psychological distress was assessed using a standardised scale comprising seven items related to positive or negative emotions, with higher scores indicating lower levels of psychological distress (possible range: 7–35 points). Dietary diversity was evaluated using a semiquantitative questionnaire including the frequency of intake of nine major food sources, with higher scores indicating higher levels of diversity (possible range: 9–27 points). Daily activity ability was assessed using the Katz Index, with scores ranging from 0 to 6; a score below 6 was classified as indicative of disability. Cognitive function was measured using the Mini-Mental State Examination (MMSE), with illiterate participants scoring below 18 and non-illiterate participants scoring below 24 considered cognitively impaired. A standardised questionnaire was employed to gather data on chronic disease history, including hypertension, diabetes, heart disease, cerebrovascular disease, and respiratory disease.

### Statistical analysis

We described continuous variables using means and standard deviations (SDs), and categorical variables as frequencies and proportions. We used analysis of variance or χ^2^ tests to compare baseline characteristics across different LAs groups. We analysed survival probabilities between groups using the Kaplan-Meier method and the Log-rank test. Further, we evaluated the association between changes in LAs and the risk of all-cause mortality using the Cox proportional hazards model, with the proportional hazard assumption tested using the Schoenfeld residuals method. Model 1 was adjusted for age and sex; model 2 was further adjusted for household registration, ethnicity, financial support, family income, living alone, illiteracy, marital status, smoking, alcohol consumption, body mass index, and diversity; model 3 was additionally adjusted for psychological distress, disability, cognitive impairment, hypertension, diabetes, heart disease, cerebrovascular disease, and respiratory disease. We used the stable moderate group as the reference to calculate hazard ratios (HRs) and 95% confidence intervals (CI) for each model. We conducted subgroup analyses stratified by sex (male, female) and age (<80, ≥80 years).

We performed sensitivity analyses to verify the robustness of the findings by excluding 1963 participants aged over 100 years; excluding 2170 deaths occurring within the first year; and excluding participants with heart disease (n = 1618) or cerebrovascular disease (n = 914) at baseline. Considering the impact of seasonal variations on changes in LAs, we further adjusted for the month of participant recruitment; to investigate the relationship between different changing patterns of LAs and mortality. We further categorised the changes in LAs into nine classifications and re-conducted the multivariate Cox regression analysis. Additionally, we examined the impact of changes in LAs on mortality risk across different follow-up periods through time stratified analyses (one year, three years, five years, and 10 years). We conducted all statistical analyses in *R*, version 4.2.0 (R Core Team, Vienna, Austria). A two-tailed *P*-value <0.05 indicated statistical significance.

## RESULTS

### Demographic characteristics

The mean age of the 21 262 older individuals was 85.16 years (SD = 11.10) and 56.06% were female. The majority of participants were of Han ethnicity (93.26%) and wer registered in rural housheolds (77.56%). In the total sample, 13.72% lived alone, 78.64% were disabled, and 15.63% had cognitive impairment. There were significant differences in the main demographic characteristics and health behaviours among the different groups of LA changes (**Table 1**).

**Table 1:** Demographic characteristics of 21 262 older adults across changes in LAs*

	Overall (n = 21 262)	Stable low (n = 4329)	Stable moderate (n = 3200)	Stable high (n = 4666)	LAs increase (n = 3919)	LAs decrease (n = 5148)	F/ꭓ^2^	*P*-value†
**Age in years, x̄ (SD)**	85.16 (11.10)	94.26 (7.64)	85.88 (9.20)	76.33 (9.43)	85.14 (10.17)	85.09 (10.36)	2028.32	<0.001
**Female**	11 919 (56.06)	3162 (73.04)	1771 (55.34)	2000 (42.86)	2082 (53.13)	2904 (56.41)	874.37	<0.001
**Han ethnic**	19 830 (93.26)	4009 (92.61)	3000 (93.75)	4356 (93.368)	3652 (93.19)	4813 (93.49)	4.64	0.325
**Rural**	16 491 (77.56)	3353 (77.45)	2534 (79.19)	3317 (71.09)	3126 (79.77)	4161 (80.83)	154.63	<0.001
**Financial support**							2880.04	<0.001
Self-supported through employment	2099 (9.87)	43 (0.99)	236 (7.38)	921 (19.74)	350 (8.93)	549 (10.66)		
Retirement wage	3579 (16.83)	272 (6.28)	442 (13.81)	1618 (34.68)	510 (13.01)	737 (14.32)		
Relative or community	15 584 (73.30)	4014 (92.72)	2522 (78.81)	2127 (45.59)	3059 (78.06)	3862 (75.02)		
**Adequate income**	14 244 (66.99)	2357 (54.45)	2150 (67.19)	3684 (78.95)	2429 (61.98)	3624 (70.40)	690.71	<0.001
**Live alone**	2870 (13.50)	335 (7.74)	521 (16.28)	582 (12.47)	523 (13.35)	909 (17.66)	237.19	<0.001
**Illiteracy**	13 018 (61.23)	3512 (81.13)	2102 (65.69)	1639 (35.13)	2497 (63.72)	3268 (63.48)	2143.65	<0.001
**Spouse alive**	6985 (32.85)	491 (11.34)	909 (28.41)	2738 (58.68)	1226 (31.28)	1621 (31.49)	2431.15	<0.001
**BMI in kg/m^2^, x̄ (SD)**	19.92 (4.03)	18.69 (3.99)	19.83 (3.85)	21.12 (4.03)	20.03 (3.98)	19.84 (3.89)	72.85	<0.001
**Smoking**	4331 (20.37)	519 (11.99)	670 (20.94)	1284 (27.52)	827 (21.10)	1031 (20.03)	350.83	<0.001
**Alcohol consumption**	4706 (22.13)	717 (16.56)	725 (22.66)	1265 (27.11)	860 (21.94)	1139 (22.13)	147.86	<0.001
**Dietary diversity, x̄ (SD)**	18.79 (2.92)	17.96 (2.94)	18.50 (2.81)	19.81 (2.82)	18.48 (2.87)	18.97 (2.82)	995.46	<0.001
**Psychological distress, x̄ (SD)**	25.02 (3.91)	23.32 (3.71)	24.77 (3.68)	26.66 (3.71)	24.51 (3.82)	25.09 (3.79)	1427.88	<0.001
**Disability**	3964(18.64)	2084 (48.14)	419 (13.09)	158 (3.39)	655 (16.71)	648 (12.59)	3160.59	<0.001
**Cognitive impairment**	3323 (15.63)	1364 (31.51)	456 (14.25)	238 (5.10)	577 (14.72)	688 (13.36)	1212.67	<0.001
**Hypertension**	3443 (16.19)	596 (13.77)	501 (15.66)	840 (18.00)	623 (15.90)	883 (17.153)	34.91	<0.001
**Diabetes**	358 (1.68)	40 (0.92)	51 (1.59)	129 (2.76)	63 (1.61)	75 (1.46)	47.63	<0.001
**Heart disease**	1618 (7.61)	250 (5.78)	225 (7.03)	451 (9.67)	291 (7.43)	401 (7.79)	50.46	<0.001
**Cerebrovascular disease**	914 (4.30)	236 (5.45)	117 (3.66)	194 (4.16)	153 (3.90)	214 (4.16)	18.32	0.001
**Respiratory disease**	2258 (10.62)	462 (10.67)	364 (11.38)	509 (10.91)	405 (10.33)	518 (10.06)	4.35	0.361

BMI – body mass index, LAs – leisure activities, SD – standard deviation, x̄ – mean

*Presented as n (%) unless specified otherwise.

†*P*-values were generated using variance analyses or χ^2^ test.

### Associations of change in total LAs with mortality risk

**Figure 2:**
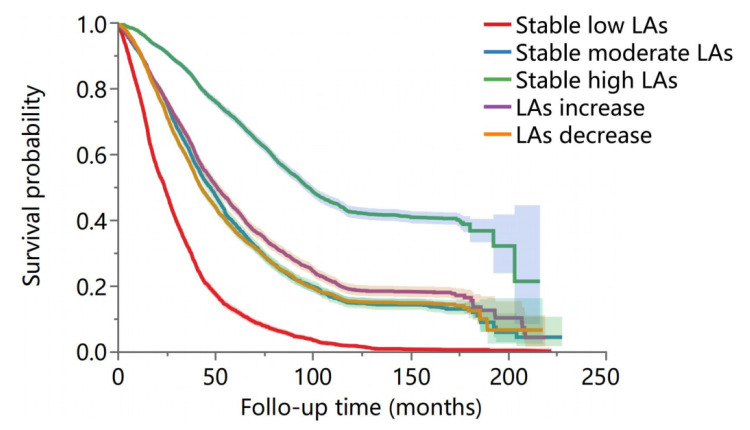
Kaplan-Meier survival curves across changing patterns of leisure activities.

A total of 15 065 deaths occurred during the follow-up period of 80 045.39 person-years. The stable low group had the highest mortality rate, at 542.79 per 1000 person-years; the stable high group had the lowest, at 79.28 per 1000 person-years. There was a statistically significant difference in survival probabilities among the different LA change characteristics among the older population (*P* < 0.001) (**Figure 2**).

After adjusting for all covariates, compared with the stable moderate group, the adjusted hazard ratio (aHR) of mortality for the stable low group and the stable high group were 1.27 (95% CI = 1.21–1.35) and 0.66 (95% CI = 0.62–0.71) in the total sample, respectively (**Table 2**). Additionally, an increase in LAs was associated with a lower risk of mortality (aHR = 0.83; 95% CI = 0.78–0.88), while a decrease in LAs was associated with a higher risk of mortality (aHR = 1.05; 95% CI = 1.01–1.09). In subgroups stratified by sex (male, female) and age (<80, ≥80 years), compared to the sustained moderate group, the stable high group had the lowest risks for all-cause mortality. An increase of LAs was associated with a lower risk of mortality (aHR = 0.76; 95% CI = 0.63–0.85), but failed to reach statistical significance in women (aHR = 0.93; 95% CI = 0.85–1.01).

**Table 2:** Associations of change in leisure activities with all-cause mortality and stratified analyses by age and sex*

				Model 1		Model 2		Model 3	
	**Participants**	**Deaths**	**Person years**	**HR (95% CI)**	***P*-value**	**HR (95% CI)**	***P*-value**	**HR (95% CI)**	***P*-value**
**Total sample**									
Stable moderate	3200	2371	10 836.81	ref		ref		ref	
Stable low	4329	3943	7264.38	1.38 (1.31–1.46)	<0.001	1.34 (1.28–1.42)	<0.001	1.27 (1.21–1.35)	<0.001
Stable high	4666	2190	27 622.63	0.66 (0.62–0.71)	<0.001	0.63 (0.59–0.66)	<0.001	0.66 (0.62–0.71)	<0.001
LAs increase	3919	2769	15 148.68	0.88 (0.83–0.94)	<0.001	0.85 (0.81–0.90)	<0.001	0.83 (0.78–0.88)	<0.001
LAs decrease	5148	3792	19 172.90	1.09 (1.03–1.15)	0.001	1.07 (1.02–1.13)	<0.001	1.05 (1.01–1.09)	0.003
**Men**									
Stable moderate	1429	1096	4413.87	ref		ref		ref	
Stable low	1167	1076	1826.43	1.33 (1.21–1.46)	<0.001	1.33 (1.22–1.45)	<0.001	1.26 (1.14–1.38)	<0.001
Stable high	2666	1351	14 783.15	0.66 (0.61–0.72)	<0.001	0.63 (0.56–0.68)	<0.001	0.65 (0.59–0.72)	<0.001
LAs increase	1837	1278	7253.96	0.79 (0.73–0.87)	<0.001	0.79 (0.71–0.84)	<0.001	0.76 (0.63–0.85)	<0.001
LAs decrease	2244	1702	8435.99	1.05 (0.96–1.13)	0.243	0.93 (0.87–1.09)	0.087	0.95 (0.88–1.03)	0.235
**Women**									
Stable moderate	1771	1275	6422.94	ref		ref		ref	
Stable low	3162	2867	5437.95	1.44 (1.33–1.55)	<0.001	1.38 (1.29–1.48)	<0.001	1.32 (1.22–1.43)	<0.001
Stable high	2000	839	12 839.48	0.65 (0.59–0.72)	<0.001	0.62 (0.57–0.67)	<0.001	0.65 (0.58–0.71)	<0.001
LAs increase	2082	1491	7894.72	0.96 (0.89–1.04)	0.345	0.94 (0.87–1.02)	0.103	0.93 (0.85–1.01)	0.065
LAs decrease	2904	2090	10 736.90	1.13 (1.05–1.21)	0.001	1.12 (1.03–1.21)	0.001	1.13 (1.07–1.19)	<0.001
**<80 y**									
Stable moderate	607	307	4115.17	ref		ref		ref	
Stable low	109	86	450.87	1.95 (1.68–2.32)	<0.001	1.92 (1.64–2.74)	<0.001	1.88 (1.41–2.32)	<0.001
Stable high	2821	933	20 876.86	0.69 (0.61–0.79)	<0.001	0.71 (0.61–0.79)	<0.001	0.76 (0.66–0.86)	<0.001
LAs increase	941	363	6446.87	0.77 (0.66–0.89)	<0.001	0.78 (0.67–0.92)	0.002	0.79 (0.68–0.91)	0.004
LAs decrease	1350	679	8717.51	1.11 (0.97–1.28)	0.114	1.12 (0.97–1.28)	0.109	1.13 (0.98–1.29)	0.075
**≥80 y**									
Stable moderate	2593	2064	6721.64	ref		ref		ref	
Stable low	4220	3857	6813.51	1.45 (1.36–1.54)	<0.001	1.40 (1.32–1.48)	<0.001	1.33 (1.25–1.42)	<0.001
Stable high	1845	1257	6745.77	0.72 (0.67–0.78)	0.001	0.70 (0.66–0.76)	<0.001	0.71 (0.65–0.76)	<0.001
LAs increase	2978	2406	8701.81	0.93 (0.87–0.99)	0.032	0.89 (0.84–0.94)	0.001	0.86 (0.81–0.92)	<0.001
LAs decrease	3798	3113	10 455.39	1.12 (1.05–1.19)	<0.001	1.11 (1.04–1.18)	<0.001	1.09 (1.02–1.17)	0.001

CI – confidence interval, HR – hazard ratios, LAs – leisure activities, ref – reference

*Model 1: Adjusted for age and sex (not in sex-subgroup analyses); Model 2: model 1 plus adjustments for ethnicity, household registration, financial support, family income, living arrangement, education level, marital status, smoking, alcohol consumption, body mass index, and dietary diversity. Model 3: model 2 plus adjustments for psychological distress, disability, cognitive impairment, hypertension, diabetes, heart disease, cerebrovascular disease, and respiratory diseases.

### Associations of change in specific activity with mortality risk

**Figure 3:**
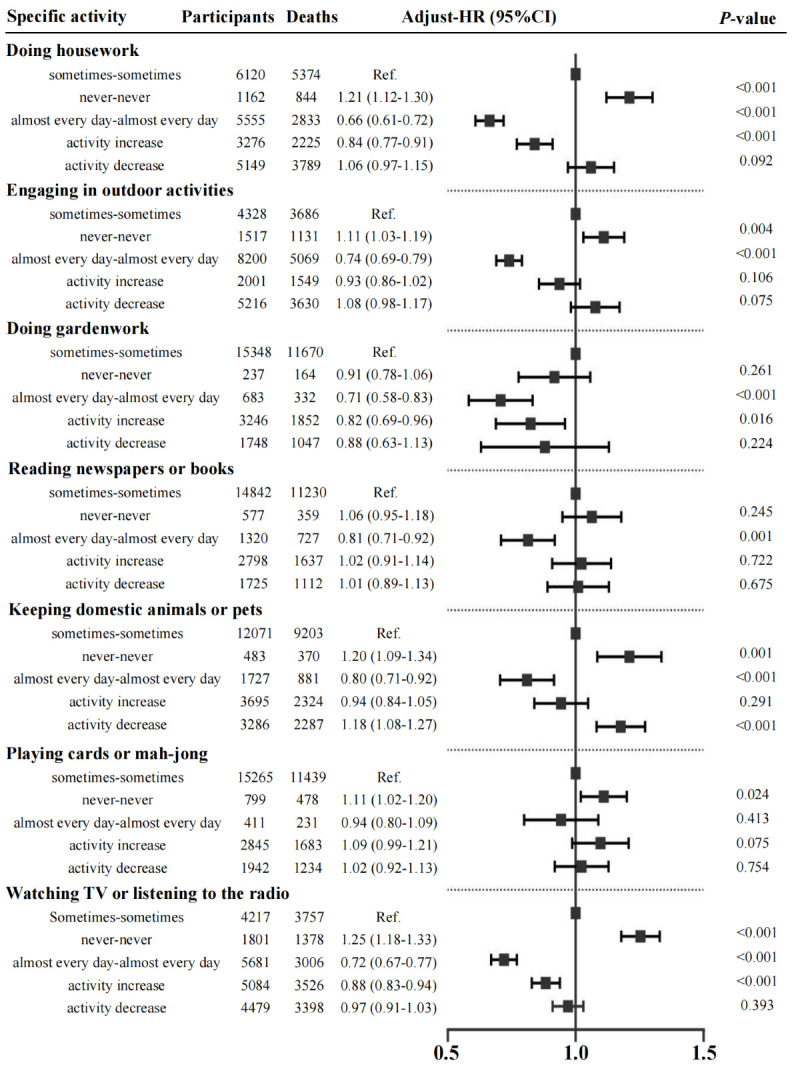
Associations of change in specific activity with mortality risk. All analyses were adjusted for age, sex, ethnicity, household registration, financial support, family income, living arrangement, education level, marital status, smoking, alcohol consumption, body mass index, dietary diversity, psychological distress, disability, cognitive impairment, hypertension, diabetes, heart disease, cerebrovascular disease, and respiratory disease.

Based on the change in each specific LA, we divided the participants five groups: group 1 (stable low: never-never), group 2 (stable moderate: sometimes-sometimes), group 3 (stable high: almost every day-almost every day), group 4 (activity increase: never-sometimes, never-almost every day, sometimes-almost every day), and group 5 (activity decrease: almost every day-sometimes, almost every day-never, sometimes-never). Using the stable moderate group (group 2) as the reference, we explored the relationship between each specific activity and all-cause mortality (**Figure 3**). For every LA examined, the stable high participation group (group 3) exhibited the lowest mortality risk. Notably, those who consistently engaged in housework experienced a 34 % reduction in mortality risk (aHR = 0.66; 95 % CI = 0.61–0.72), representing the greatest decrease among all activities. Regular gardening was also associated with a substantial 29 % reduction in mortality odds (aHR = 0.71; 95 % CI = 0.58–0.83). Moreover, participants whose involvement increased over time in housework (aHR = 0.84; 95 % CI = 0.77–0.91), gardening (aHR = 0.82; 95 % CI = 0.69–0.96), or television viewing/listening to the radio (aHR = 0.88; 95 % CI = 0.83–0.94) likewise demonstrated significantly lower risks of death 

For each LA, the group with stable high participation (group 3) had the lowest risk of death. Additionally, we found that increased participation in housework (aHR = 0.84; 95% CI = 0.77–0.91), gardening (aHR = 0.82; 95% CI = 0.69–0.96), and watching TV or reading to the radio (aHR = 0.88; 95% CI = 0.83–0.94) significantly reduced the risk of death.

### Sensitivity analyses

The sensitivity analyses did not substantially alter the main findings (Table S2–5 in the **Online Supplementary Document**). Stable high LAs and LAs increase groups were associated with lower mortality risk. Exclusion of participants aged >100 years, those with cerebrovascular disease, or heart disease did not substantially alter the association between change in LAs and all-cause mortality. When excluding older participants who died in the first year, the HRs of all-cause mortality for change in LAs showed slight reduction, but the overall conclusions remained robust. Besides, when we further considered the months of sample recruitment, maintaining and improving LAs could still significantly reduce the risk of all-cause mortality (Table S6 in the **Online Supplementary Document**).

When changes in LAs were included as a nine-categories variable in the fully adjusted model, the stable high group remained associated with the lowest mortality risk, while the stable low group continued to be associated with the highest mortality risk (Table S7 in the **Online Supplementary Document**). Regarding the different patterns of LAs increase, we found that the mortality risk in the slow increase group (low to moderate) did not decrease (HR = 0.94; 95% CI = 0.88–1.02). However, the sharp increase group (low to high) was associated with a lower mortality risk (HR = 0.80; 95% CI = 0.72–0.90). Additionally, we found that the HRs remained significant and comparable for both short-term (one year and three years) and long-term mortality (five years and 10 years) in the Cox regressions stratified by different follow-up periods, indicating a robust effect of changes in LAs on overall mortality across time (Table S8 in the **Online Supplementary Document**).

## DISCUSSION

Using data from a large, representative cohort of older Chinese adults, we showed that active participation in LAs was linked to a reduced risk of mortality. Compared with the stable moderate group, the HR of mortality was higher for the stable low group and lower for the stable high group. Moreover, increased participation in LAs corresponded with a reduced mortality risk. These findings broaden our understanding of the dynamic relationship between LAs and mortality, emphasising the importance of sustained, high-level participation in LAs for promoting health among community-dwelling older adults.

Western societies have made significant progress in helping older adults meet national recommendations for LAs, with emerging studies indicating that frequent participation is associated with reduced all-cause mortality among the older population. The seven LAs assessed in this study are more culturally relevant and tailored to the characteristics of older adults in Chinese communities. Furthermore, these activities are simple and easy to perform, making them well-suited for promoting longevity in this population. The potential explanation for this association lies in the multifaceted impact of LAs on health and well-being, driven by a complex interplay of psychological, biological, social, and behavioural mechanisms [23]. Psychologically, LAs enhance mood, emotional well-being, and foster a sense of purpose, all of which may contribute to a reduced risk of mortality [24]. Biologically, such activities stimulate the nervous system, regulate hormone levels, and improve cardiometabolic health, thereby positively influencing longevity [25,26].

From a social perspective, participation in LAs promote increased social engagement and support, strengthening networks that are well-established to improve health outcomes. Behaviourally, LAs engagement encourages the adoption of healthier habits while reducing engagement in harmful behaviours, directly impacting disease risk and mortality [27]. Furthermore, examining LAs through the lens of complex adaptive systems emphasises the interactions among these factors, showing that the collective impact of LAs on health exceeds the sum of individual components. This holistic perspective highlights the multiple pathways through which LAs can lead to lower all-cause mortality. After further adjusting for multiple health and disease conditions, the HR for mortality was partially attenuated (**Table 2**). We also found that higher LAs scores at baseline were associated with better activities of daily living, cognitive performance, and mental health status. This finding suggests that the impact of changes in LAs on mortality may be explained through physical activity, cognitive function, and comorbidities. Future research is needed to further explore their potential mediating effects on LAs.

Consistent with the findings of Li et al. [4] and Agahi et al. [28], we observed that increased participation in LAs, such as doing housework, gardening, watching TV or listening to the radio, was associated with lower mortality risk after full adjustment (**Figure 3**). Engaging in housework involves daily tasks that are accessible and cost-effective for older people, since this activity does not require professional equipment and facilities. Doing housework helps older individuals maintain muscle strength, physical fitness, and body flexibility [29] and contributes to better cognitive performance, such as improved memory and mental agility [30]. At the interpersonal level, activities like cooking for the family or taking care of grandchildren can enhance social interaction and reduce feelings of isolation [31]. Engaging in outdoor recreation or gardening can enhance the mobility of older adults. These activities aid in maintaining flexibility and boost exposure to vitamin D [32]. Outdoor activities offer a low-impact form of exercise that can mitigate the risk of chronic diseases and improve overall fitness [33,34]. However, gardening often necessitates ample open space, which can be a challenge for older adults residing in urban areas. For such individuals, container gardening or participation in community gardens can serve as effective alternatives. These options not only promote physical activity, but also foster social interactions among older participants. Therefore, it is recommended that future interventions focus on creating open activity areas or community gardens in both urban and rural regions and provide guidance for older adults.

Research on specific activities like TV watching has produced mixed results. One study found it may reduce mortality risk in older Chinese adults [35], while others reported that prolonged TV watching increases all-cause mortality risk [36,37]. A recent meta-analysis identified a J-shaped relationship between TV viewing and mortality in the general population [38], likely due to variations in studied populations, TV viewing habits, and sedentary behaviour. Several studies have also highlighted the positive effects of activities such as reading [39–42], playing board games (mahjong, chess or poker) [43,44], and playing cards [40,45] on cognitive health, which significantly reduces mortality risk. Playing mahjong or cards, in particular, fosters social engagement, known to prevent cognitive decline [46,47] and may also influence immune function and inflammatory processes [48,49]. These findings further emphasise the importance of culturally relevant LAs that support not only physical health but also cognitive and social well-being in older adults.

We further observed that among the oldest-old population (≥80 years), compared to the sustained moderate group, both the stable high and LAs increase groups exhibited lower risks for all-cause mortality, while the LAs decrease group was consistently associated with a higher mortality risk. These findings suggest that frequent engagement in LAs may exert a protective effect and reduce the risk of all-cause mortality in later life. Previous prospective studies on younger adults have generally reported protective associations between various types of LAs and mortality risk, although the results have not always been statistically significant [50,51]. Our analysis builds on this existing research by focusing on a larger sample of Chinese older adults with a longer follow-up period, revealing a universal benefit of physical activity for older adults. This study also had greater statistical power to investigate the nuanced associations between specific activities and mortality risk.

In this study, the reduction in mortality risk associated with increased LAs was more pronounced among males than females. Earlier research on gender differences in the association between LAs in old age and survival has been mixed. Some studies reported stronger associations between LAs and mortality for women than for men [28], while others, consistent with our findings, showed that the protective effects of LAs were more significant for men. For instance, Talbot and colleagues found that in men, larger declines in total and high-intensity LAs were independent predictors of all-cause mortality, whereas in women, changes in LA levels were not significant predictors of mortality [22]. Liu and colleagues’ research found stronger associations between reading books and all-cause mortality among men but not women [52], suggesting that social activities may be more beneficial for women, while solitary activities may benefit men more [53]. Social activities provide individuals with meaningful roles, larger social networks, and various forms of social support, all of which may improve coping abilities and health behaviours, ultimately protecting against adverse physiological responses. Social engagement is also strongly associated with reduced mortality, as it helps decrease risky behaviours and enhances resistance to disease [54]. Since men have traditionally had more active work lives than women, the additional benefit of active leisure time through social participation may be limited. However, activities that are particularly beneficial for men, such as gardening and hobby-related activities, tend to be solitary, voluntary, and creative/productive in nature. These activities may serve as coping strategies by reducing stress and fostering a sense of well-being, thus positively influencing survival.

This is the first study to examine the association between changes in overall and specific LAs and all-cause mortality among older adults, using repeated measures over time to capture detailed behavioural patterns. The use of simple, consistent questionnaires and a large, nationally representative sample, along with robust sensitivity analyses, strengthens the findings, offering insights for targeted interventions and minimising biases, thus enhancing the generalisability of our results. However, several limitations should be considered. First, although we adjusted for numerous health-related covariates, the potential for residual confounding, such as medication use and genetic risk, cannot be completely excluded. Second, the sample consisted exclusively of community-dwelling Chinese older adults, most of whom had rural household registration, which may limit the generalisability to older individuals in urban areas and Western countries. Therefore, caution is warranted when applying these results to other populations. Third, we assessed only a limited number of LAs by self-report questionnaire, and did not collect detailed information on the duration, intensity, or energy expenditure of each activity, which may have resulted in recall bias and imprecise measurements. Future research needs to further investigate the impact of objective activity measurements based on digital devices (such as accelerometers) on health outcomes. Finally, the observed longitudinal relationship may be influenced by ‘reverse causality’, where a decline in LAs precedes death due to underlying chronic conditions. To minimize this potential bias, we performed a sensitivity analysis, excluding participants who died within the first year of follow-up, those over 100 years of age, and individuals with heart or cerebrovascular diseases. Our findings remained consistent after these exclusions.

## CONCLUSIONS

In this large-scale longitudinal study, we found that sustaining or increasing participation in LAs significantly lowers the risk of all-cause mortality among older adults living in the community. Particularly, regular involvement in specific activities like housework, gardening, and watching TV or listening to the radio was linked to a substantial reduction in premature death.

## Additional material


Online Supplementary Document
