# Supplementary material for: Association of changes in overall and specific leisure activities with long-term all-cause mortality in older adults: a nationwide cohort study
Source: J Glob Health. 2025 May 16;15:04119. doi: 10.7189/jogh.15.04119 (PMC12082748; doi:10.7189/jogh.15.04119)
Supplement: Online Supplementary Document [file jogh-15-04119-s001.pdf]

Supplement to: Zhan C, Bai A, Kang Y, Zhang Y, Zhao J, Shi H, Shen J. Association of changes in overall and specific leisure activities with long-term all-cause mortality in older adults: a nationwide cohort study. J Glob Health. 2025;15:04119.

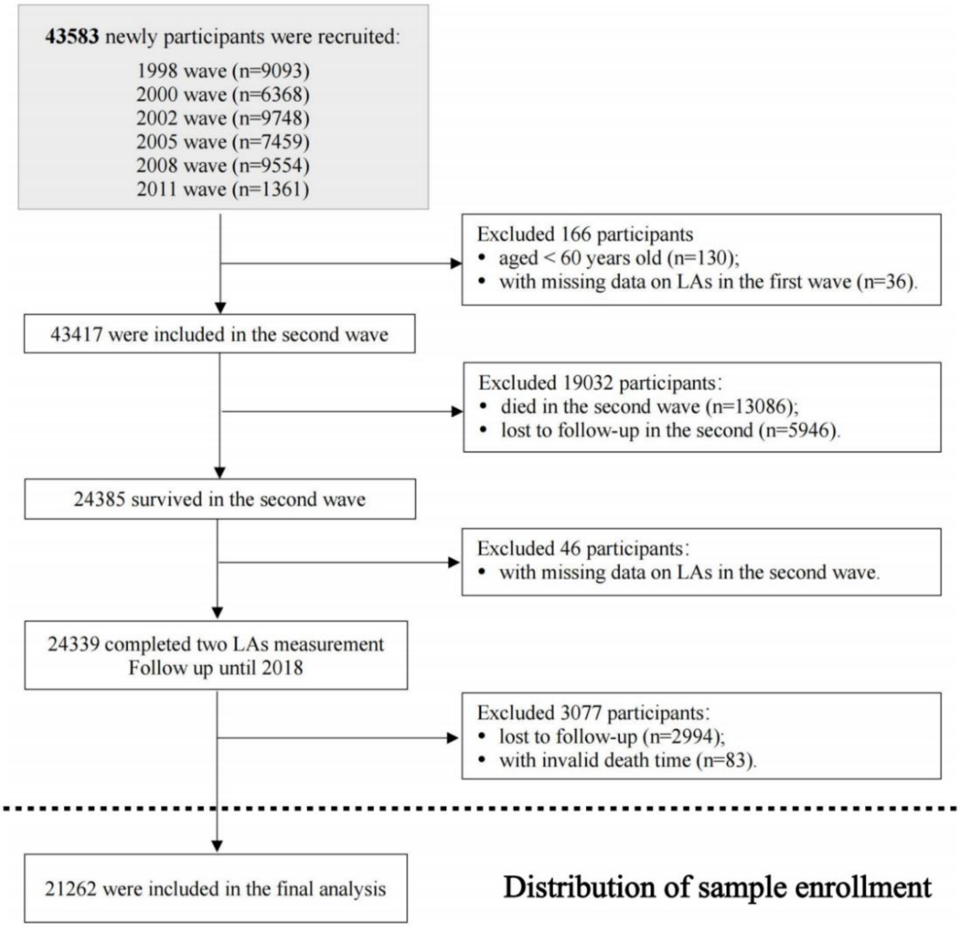

Wave		1998	2000	2002	2005	2008	2011	2014	2018	Participants	Deaths
**1998 wave**	LAs measurement	T1	T2							4186	3609
	Follow-up			→	→	→	→	→	→		
**2000 wave**	LAs measurement		T1	T2						3895	3327
	Follow-up				→	→	→	→	→		
**2002 wave**	LAs measurement			T1	T2					4677	3051
	Follow-up					→	→	→	→		
**2005 wave**	LAs measurement				T1	T2				2944	1930
	Follow-up						→	→	→		
**2008 wave**	LAs measurement					T1	T2			4786	2835
	Follow-up							→	→		
**2011 wave**	LAs measurement						T1	T2		774	313
	Follow-up								→		

Figure S1. Inclusion and exclusion process of study participants.

Table S1. Details of the 7-item leisure-time activities engagement scale

Item	Content	Options
1	Doing housework	almost every day=2; sometimes=1; rarely/never=0
2	Engaging in outdoor activities	almost every day=2; sometimes=1; rarely/never=0
3	Doing gardenwork	almost every day=2; sometimes=1; rarely/never=0
4	Reading newspapers or books	almost every day=2; sometimes=1; rarely/never=0
5	Keeping domestic animals or pets	almost every day=2; sometimes=1; rarely/never=0
6	Playing cards or mah-jong	almost every day=2; sometimes=1; rarely/never=0
7	Watching TV or listening to the radio	almost every day=2; sometimes=1; rarely/never=0

Table S2. Associations of change in leisure activities with all-cause mortality after excluding 1963 participants aged >100 years

19299 participants were included

Groups	Participant	Deaths	Person years	Fully adjusted model	
				HR (95% CI)	*P*-value
Stable moderate	3007	2191	10589.43	Ref.	
Stable low	3242	2922	6028.48	1.25 (1.19-1.33)	<0.001
Stable high	4611	2146	27528.4	0.64 (0.58-0.69)	<0.001
LAs increase	3666	2541	14639.74	0.84 (0.79-0.90)	<0.001
LAs decrease	4773	3451	18437.95	1.05 (1.01-1.08)	0.002

Note: Adjusted for age, sex, ethnicity, household registration, financial support, family income, living arrangement, education level, marital status, smoking, alcohol consumption, body mass index, dietary diversity, psychological distress, disability, cognitive impairment, hypertension, diabetes, heart disease, cerebrovascular disease, and respiratory disease. LAs, leisure activities; HR, hazard ratios; CI, confidence interval.

Table S3. Associations of change in leisure activities with all-cause mortality after excluding 2170 participants died in the first year

19092 participants were included

Groups	Participant	Deaths	Person years	Fully adjusted model	
				HR (95% CI)	*P*-value
Stable moderate	2909	2080	10685.86	Ref.	
Stable low	3469	3083	6812.55	1.19 (1.06-1.32)	<0.001
Stable high	4515	2039	27535.55	0.68 (0.64-0.74)	<0.001
LAs increase	3559	2409	14963.81	0.81 (0.74-0.88)	<0.001
LAs decrease	4640	3284	18876.86	1.07 (1.02-1.12)	0.004

Note: Adjusted for age, sex, ethnicity, household registration, financial support, family income, living arrangement, education level, marital status, smoking, alcohol consumption, body mass index, dietary diversity, psychological distress, disability, cognitive impairment, hypertension, diabetes, heart disease, cerebrovascular disease, and respiratory disease. LAs, leisure activities; HR, hazard ratios; CI, confidence interval.

Table S4. Associations of change in leisure activities with all-cause mortality after excluding 1618 participants with heart disease

19644 participants were included

Groups	Participant	Deaths	Person years	Fully adjusted model	
				HR (95% CI)	*P*-value
Stable moderate	2975	2209	10200.55	Ref.	
Stable low	4079	3719	6939.97	1.29 (1.22-1.37)	<0.001
Stable high	4215	1997	25016.01	0.67 (0.61-0.72)	<0.001
LAs increase	3628	2592	14048.04	0.82 (0.78-0.86)	<0.001
LAs decrease	4747	3502	17812.2	1.06 (1.02-1.11)	0.002

Note: Adjusted for age, sex, ethnicity, household registration, financial support, family income, living arrangement, education level, marital status, smoking, alcohol consumption, body mass index, dietary diversity, psychological distress, disability, cognitive impairment, hypertension, diabetes, heart disease, cerebrovascular disease, and respiratory disease. LAs, leisure activities; HR, hazard ratios; CI, confidence interval.

Table S5. Associations of change in total leisure activities with all-cause mortality after excluding 914 participants with cerebrovascular disease

20348 participants were included

Groups	Participant	Deaths	Person years	Fully adjusted model	
				HR (95% CI)	*P*-value
Stable moderate	3083	2295	10413.6	Ref.	
Stable low	4093	3730	7005.29	1.23 (1.17-1.30)	<0.001
Stable high	4472	2102	26547.72	0.67 (0.64-0.71)	<0.001
LAs increase	3766	2664	14653.59	0.82 (0.75-0.90)	<0.001
LAs decrease	4934	3636	18455.02	1.05 (1.02-1.08)	0.002

Note: Adjusted for age, sex, ethnicity, household registration, financial support, family income, living arrangement, education level, marital status, smoking, alcohol consumption, body mass index, dietary diversity, psychological distress, disability, cognitive impairment, hypertension, diabetes, heart disease, cerebrovascular disease, and respiratory disease. LAs, leisure activities; HR, hazard ratios; CI, confidence interval.

Table S6. Associations of change in total leisure activities with all-cause mortality after additionally adjusting for the month of participant recruitment

Groups	Participant	Deaths	Person years	Fully adjusted model	
				HR (95% CI)	*P*-value
Stable moderate	3200	2371	10836.81	Ref.	
Stable low	4329	3943	7264.38	1.26 (1.16-1.29)	<0.001
Stable high	4666	2190	27622.63	0.67 (0.63-0.72)	<0.001
LAs increase	3919	2769	15148.68	0.83 (0.77-0.89)	<0.001
LAs decrease	5148	3792	19172.90	1.07 (1.03-1.11)	0.001

Note: Adjusted for age, sex, ethnicity, household registration, financial support, family income, living arrangement, education level, marital status, smoking, alcohol consumption, body mass index, dietary diversity, psychological distress, disability, cognitive impairment, hypertension, diabetes, heart disease, cerebrovascular disease, respiratory disease, and the month of recruitment.
LAs, leisure activities; HR, hazard ratios; CI, confidence interval.

Table S7. Associations of nine categories of changes in LAs with all-cause mortality

Groups	Participants, n	Death, n (%)	Person-years	HR (95%CI)	*P*-value
low - low	4329	3943	17437.53	1.25(1.18-1.33)	<0.001
low - moderate	1542	1286	7878.22	0.94(0.88-1.02)	0.131
low - high	579	443	3261.55	0.80(0.72-0.90)	<0.001
moderate - low	2041	1730	10082.68	1.15(1.07-1.23)	<0.001
moderate - moderate	3200	2371	18356.81	Ref	
moderate - high	1798	1040	13218.55	0.70(0.65-0.75)	<0.001
high - low	933	760	5424.16	1.11(1.02-1.20)	0.021
high - moderate	2174	1302	15763.86	1.04(0.96-1.13)	0.237
high - high	4666	2190	38587.73	0.61(0.58-0.65)	<0.001

Note: Adjusted for age, sex, ethnicity, household registration, financial support, family income, living arrangement, education level, marital status, smoking, alcohol consumption, body mass index, dietary diversity, psychological distress, disability, cognitive impairment, hypertension, diabetes, heart disease, cerebrovascular disease, and respiratory disease. LAs, leisure activities; HR, hazard ratios; CI, confidence interval.

Table S8. Associations of changes in LAs with short-term and long-term all-cause mortality stratified by different follow-up periods

Groups	Participants	Deaths	HR (95% CI)	*P*-value
**1-year mortality**				
Stable moderate	3200	291	Ref.	
Stable low	4329	860	1.32 (1.07-1.63)	0.008
Stable high	4666	151	0.52 (0.38-0.70)	<0.001
LAs increase	3919	360	0.78 (0.62-0.96)	0.023
LAs decrease	5148	508	1.02 (0.83-1.30)	0.624
**3-years mortality**				
Stable moderate	3200	1389	Ref.	
Stable low	4329	3130	1.34 (1.23-1.47)	<0.001
Stable high	4666	796	0.65 (0.58-0.72)	<0.001
LAs increase	3919	1550	0.92 (0.87-0.97)	0.002
LAs decrease	5148	2245	1.08 (1.03-1.13)	0.016
**5-years mortality**				
Stable moderate	3200	1923	Ref.	
Stable low	4329	3688	1.28 (1.18-1.37)	<0.001
Stable high	4666	1348	0.68 (0.63-0.74)	<0.001
LAs increase	3919	2190	0.92 (0.85-0.98)	0.026
LAs decrease	5148	3128	1.06 (1.02-1.11)	0.003
**10-years mortality**				
Stable moderate	3200	2351	Ref.	
Stable low	4329	3924	1.27 (1.19-1.36)	<0.001
Stable high	4666	2157	0.71 (0.65-0.78)	<0.001
LAs increase	3919	2749	0.88 (0.81-0.95)	<0.001
LAs decrease	5148	3779	1.04 (1.01-1.07)	0.004

Note: Adjusted for age, sex, ethnicity, household registration, financial support, family income, living arrangement, education level, marital status, smoking, alcohol consumption, body mass index, dietary diversity, psychological distress, disability, cognitive impairment, hypertension, diabetes, heart disease, cerebrovascular disease, and respiratory disease. LAs, leisure activities; HR, hazard ratios; CI, confidence interval.
